# On the generalization of tones: A detailed exploration of non-speech auditory perception stimuli

**DOI:** 10.1038/s41598-020-63132-2

**Published:** 2020-06-12

**Authors:** Michael Schutz, Jessica Gillard

**Affiliations:** 1 School of the Arts, McMaster University, Hamilton, Canada; 2 Department of Psychology, Neuroscience & Behaviour, McMaster University, Hamilton, Canada

**Keywords:** Auditory system, Perception, Sensory processing

## Abstract

The dynamic changes in natural sounds’ temporal structures convey important event-relevant information. However, prominent researchers have previously expressed concern that non-speech auditory perception research disproportionately uses simplistic stimuli lacking the temporal variation found in natural sounds. A growing body of work now demonstrates that some conclusions and models derived from experiments using simplistic tones fail to generalize, raising important questions about the types of stimuli used to assess the auditory system. To explore the issue empirically, we conducted a novel, large-scale survey of non-speech auditory perception research from four prominent journals. A detailed analysis of 1017 experiments from 443 articles reveals that 89% of stimuli employ amplitude envelopes lacking the dynamic variations characteristic of non-speech sounds heard outside the laboratory. Given differences in task outcomes and even the underlying perceptual strategies evoked by dynamic vs. invariant amplitude envelopes, this raises important questions of broad relevance to psychologists and neuroscientists alike. This lack of exploration of a property increasingly recognized as playing a crucial role in perception suggests future research using stimuli with time-varying amplitude envelopes holds significant potential for furthering our understanding of the auditory system’s basic processing capabilities.

## Introduction

When designing research studies, scientists strive to minimize confounds potentially confusing experimental outcomes. The most famous cautionary tale of failing to control for extraneous variables can be found in Hans the counting horse, who delighted early 20^th^ century audiences by appearing to answer basic arithmetic questions through sequential taps of his hoof. Subsequent investigation revealed the true source of his seemingly remarkable talent—rather than calculating, ‘Clever Hans’ merely recognized the reactions of humans who moved with excitement after seeing the correct number of taps^1^. Although disappointing for his fans, it provided such an invaluable lesson in experimental control that it is still routinely discussed in introductory psychology textbooks^2,3^—a century after Hans’s debut.

Today, researchers take great pains to avoid confounding factors through carefully designed paradigms employing tightly controlled stimuli. Although this approach has undoubtedly contributed to psychology’s success in explaining many complex phenomena, overuse of simplified tones in experiments can lead to inaccurate perspectives on perceptual processing. Here we examine this issue of broad importance through an in-depth study of the stimuli used to assess non-speech auditory perception, an exploration holding important implications for interpreting a wide body of perceptual research.

## Controlled auditory stimuli

Sounds synthesized with temporal shapes (“amplitude envelopes”) consisting of rapid onsets followed by sustain periods and rapid offsets afford precise quantification and description—qualities of obvious methodological value. However as William Gaver argued in a different context, fixating on simplistic sounds can lead researchers astray when attempting to explore the processes used in everyday listening^4,5^. For example, a sound’s amplitude envelope is rich in information, allowing listeners to discern the materials involved in an event^6,7^, or even an event’s outcome—such as whether a dropped bottle bounced or broke^8^. However, this cue is largely absent in synthesized tones with abrupt offsets, as their short decays provide no information about sound-producing events and materials. Therefore the simplistic structures of tone beeps, buzzes, and clicks do not necessarily trigger the same perceptual processes as natural sounds—potentially complicating attempts to generalize from experimental outcomes to our processing of sounds outside the laboratory.

The ecological relevance of auditory stimuli outside of speech has ironically grown more problematic as the field evolves. Early experiments employed natural sounds such as balls dropping on hard surfaces and hammers striking plates^9^. However with the invention of the vacuum tube and then modern computers, many researchers eagerly traded natural sounds for precisely controlled tones^10^. Concern with this decision is hardly novel, as colleagues have previously expressed worry that much of auditory psychophysics “lack[s] any semblance of ecological validity”^10^ given the dearth of amplitude invariant (i.e. “flat”) tones in the natural world^11^. Although some have articulated the merits of using stimuli with more varied amplitude envelopes^12^, to the best of our knowledge there has been no large-scale formal exploration of non-speech auditory perception stimuli—a useful step in understanding the current state of the field so as to improve future approaches.

### Amplitude Envelope’s Crucial Role in Perceptual Organization

Although amplitude envelope’s importance in timbre is widely recognized^13–15^, its role in other perceptual constructs and processes has often received less attention. Consequently many experiments are conducted with a single type of amplitude envelope—the temporally simplistic flat tone. Their artificial characteristics embody the concern clearly articulated by Gaver^4,5^ and others^10^ warning of a divide between the auditory system’s use in everyday listening and its assessment in the laboratory. The following series of experiments on audio-visual integration illustrates one specific example of problems endemic with over-using a single type of stimulus to pursue a generalized understanding of psychological processes.

**Figure 1:**
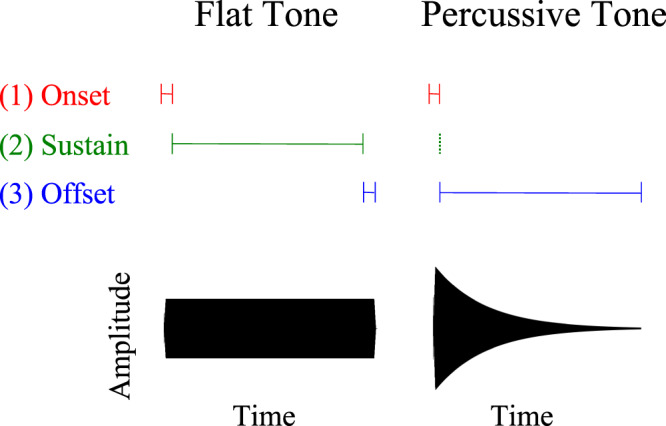
Flat and percussive amplitude envelopes. The rapid onset segment (1) is often similar in flat and percussive tones. The sustain segment (2) constitutes a large percentage of a flat tones, but is non-existent in percussive tones. Conversely the offset segment (3) is typically brief in flat tones whereas it constitutes the majority of percussive tones.

Videos of a renowned musician using long and short striking movements illustrate that vision can strongly affect judgments of musical note duration^16^. This illusion persists when using impact (but not sustained) sounds from other events^17^, point-light simplifications of the movements^18^, and even a single moving dot^19^. Curiously however, it breaks with widely-accepted thinking that vision exerts little influence on auditory judgments of event duration^20–22^. This conflict has its roots in the dynamically decaying amplitude envelope (i.e. “sound shape”) of sounds created by natural impacts such as those produced by the marimba (Fig. 1). Further explorations demonstrate that pure tones shaped with the amplitude envelopes characteristic of impacts integrate with visual information, whereas the same pure tones shaped with flat amplitude envelopes (i.e., traditional “beeps”) do not^23^. This illustrates that conclusions derived from experiments with flat tones do not necessarily generalize to real-world tasks, as their simplified temporal structures fail to trigger the same perceptual processes as natural sounds.

Amplitude envelope’s effect on audio-visual integration can be seen in other tasks. For example, a click simultaneous with two disks overlapping after moving across a screen increases the probablity of perceiving a ‘bounce’ rather than the circles passing through one another^24^. However, damped tones (i.e. decreasing in intensity over time) elicit stronger bounce percepts than ramped tones (i.e. increasing in intensity over time), presumably as they are event-consistent^25^. These two studies illustrate that in addition to amplitude envelope affecting vision’s influence on audition^16,17^ it can affect audition’s influence on vision^25^.

Repeated findings of amplitude envelope’s role in audio-visual integration^17,23,25,26^ complement a growing body of work on differences in the processing of tones with rapid increases vs. decreases in intensity (i.e., “ramped” or “looming” vs. “damped” or “receding”) in auditory processing. Although merely time-reversed and therefore spectrally matched, these sounds are perceived as differing in duration^27–31^, loudness^32–34^, and loudness change^35,36^. These observations of differences in the perception of tones distinguished only by amplitude envelope shape raise questions about whether the disproportionate use of flat tones as experimental stimuli could lead to broader problems with generalization. For example, the durations of amplitude invariant tones can be evaluated using a ‘marker strategy’—marking tone onset and offset. This approach is consistent with Scalar Expectancy Theory (SET), a widely accepted timing framework^37,38^. However such a strategy would be problematic for sounds with decaying offsets, as their moment of acoustic completion is ambiguous (Fig. 1).

### What sounds are used in auditory perception research?

In order to explore the types of stimuli used to study non-speech auditory perception, we analyzed a representative sample of experiments drawn from several decades of four well-respected journals (two focused on general psychological research, and two with a specific auditory focus). This approach builds on our team’s previous survey of *Music Perception*, which revealed surprisingly that over one-third of its studies omitted definition of amplitude envelope^39^. That survey focused heavily on musical stimuli and examined only experiments using single tones or isolated series of tones. Furthermore, it drew unequally from different time periods, making it difficult to discern trends. In order to broaden our approach, here we conducted a survey (a) exploring a variety of non-speech auditory perception tasks, (b) incorporating diverse paradigms, (c) assessing multiple stimulus properties (i.e. spectral structure, duration), and (d) involving multiple journals widely recognized for their rigor and prestige. Consequently, this project offers useful insight into sounds used to explore the auditory system—the stimuli upon which numerous theories of perceptual processing are built.

## Methods

In order to obtain a representative sample of experiments we used databases indexing articles in four highly regarded journals regularly publishing auditory perception research on human subjects. We initially began with two journals focused on general psychological processing: *Attention*, *Perception & Psychophysics* (henceforth referred to as APP) and *Journal of Experimental Psychology: Human Perception & Performance* (JEP)—both of which are indexed by *PsycInfo*. Later when expanding the survey to include the auditory-focused *Hearing Research (*HR) we turned to *Web of Science*, as HR is not indexed by *PsycInfo*. Although adequate for HR, *Web of Science* only indexes *Journal of the Acoustical Society of America* (JASA) back to 1976. Therefore we used *Web of Science* for articles published in or after 1976 to align as much as possible with our approaches to HR, and used *JASA Portal* for earlier articles.

### Selection of articles to classify

Differences in each journal’s scope necessitated slightly different search terms in order to obtain a consistent focus. For example, although our searches of APP and JEP naturally resulted in papers focused on human participants, an equivalent focus in HR required filtering out non-human animal studies. Similarly, whereas the wide range of psychophysical studies in APP and JEP necessitated use of the search term “audition”, this was unnecessary for JASA. However, JASA’s broad acoustical focus, including issues such as underwater sound transmission^40,41^ instead compelled use of “psychophysic*”—a term obviously unnecessary for APP. Complete terms used are displayed in Table 1.

**Table 1:** Summary of Search Terms.

Journal	Database	Search Terms
APP (1966–2017)	PsycInfo	Attention, Perception & Psychophysics (Publication Name) AND Auditory (Identifier) NOT Speech (Identifier) NOT Language (Identifier) NOT Phonetic (Identifier) NOT Dialect (Identifier)
JEP (1975–2017)	PsycInfo	Journal of Experimental Psychology: Human Perception & Performance (Publication Name) AND Auditory (Identifier) NOT Speech (Identifier) NOT Language (Identifier) NOT Phonetic (Identifier) NOT Dialect (Identifier) NOT Word (Identifier)
HR (1978–2017)	Web of Science	Hearing Research (Publication Name) AND Auditory (Topic) NOT Speech (Topic) NOT Language (Topic) NOT Phonetic (Topic) NOT Dialect (Topic) Article (Document Type) Social Sciences & Arts Humanities (Categories) EXCLUDE Zoology (Subject Area) EXCLUDE Veterinary Sciences (Subject Area) EXCLUDE Plant Sciences (Subject Area) EXCLUDE Agriculture (Subject Area) EXCLUDE Food Science Technology (Subject Area)
JASA (1950–1975)	JASA Portal	Psychophysic* (full bibliographic record) NOT Speech (abstract/title/keyword) NOT Animal (abstract/title/keyword)
JASA (1976–2017)	Web of Science	Journal of the Acoustical Society of America (Publication Name) AND Psychophysic* (topic) NOT Animal (topic) NOT Speech (topic)

This process resulted in a pool of 4622 potential articles. In order to select a manageable number we used a stratified quota sampling technique^42^, taking the first two to four articles per journal per year. This balanced competing desires for a sample representative of that journal’s history and rough equivalence in the number of articles per journal. For example, we selected a maximum of two articles per year from JASA (dating back to 1950), but up to four per year for JEP (established in 1975). Adapted for our purposes based on best practices for accurate sampling in public opinion polls and market research^43^, this approach yielded a final corpus of 443 papers split relatively evenly amongst the four journals (see Table 2).

**Table 2:** Summary of Article Selection and Number of Experiments.

Journal	Article Pool (N)	Selection	Sample (N)	Experiments (N)
APP (1966–2017)	466	First 2 articles from each year	104	228
JEP (1975–2017)	210	First 4 articles from each year	113	392
HR (1978–2017)	1820	First 3 articles from each year involving human subjects	114	180
JASA (1950–2017)	2126	First 2 articles from each year	112^a^	217
Total			443	1017

^a^Web of Science returned 2005 articles (1975–2017), and the JASA Portal 121 (1950–1975).

### Analysis and classification of individual experiments

We coded all experiments (n = 1017) individually within the 443 articles, classifying only the auditory components of multisensory stimuli. Due to the diversity of designs encountered, we fractionally distributed one point amongst all sound categories within each experiment—refining our team’s earlier approaches. For example, if an experiment used two sound categories (i.e. a target and distractor), each sound category received a half point. In an experiment with four types of targets and two types of distractors, each target and distractor received 0.125 and 0.25 points respectively (sample point weightings appear in Table 3). This avoided over-emphasizing individual experiments using a large number of stimuli—such as the 64 different sounds employed by Gygi and Shafiro (2011).

**Table 3:** Examples of Point Weighting Distributions.

Article	Exp. #	Sound Categories	Functional Category	Point Weight	Envelope Category
Pfordresher, 2008^135^ *Three experiments*, *each using a* *single sound*	1	1	auditory feedback	1.00	percussive
	2	1	auditory feedback	1.00	percussive
	3	1	auditory feedback	1.00	percussive
Kirby, Browning, Brennan, Spratford, & McCreery, 2015^136^ *One experiment using two sounds*	1	2	reference	0.50	undefined
			target	0.50	undefined
Stilp, Alexander, Kiefte, & Kluender, 2010^82^ *Two experiments*, *each using three types* *of sounds*	1	3	target A	0.333	other
	1		target B	0.333	other
	1		precursor	0.333	other
	2	3	target A	0.333	other
	2		target B	0.333	other
	2		precursor	0.333	other
Møller & Jho, 1989^137^ *One experiment*, *using three types of* *sounds*	1	3	signal A	0.111	flat
			signal A	0.111	flat
			signal A	0.111	flat
			signal B	0.167	click train
			signal B	0.167	click train
			masker	0.333	undefined

Note: Each experiment received a single point, which we distributed equally amongst the functional categories of the sounds used.

### Classification of Amplitude Envelope

**Figure 2:**
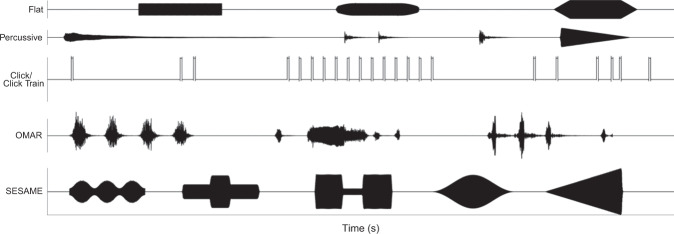
Examples of defined amplitude envelope categories: (**a**) various *Flat* tones, (**b**) *Percussive* sounds including a bell, hand claps, and bongo as well as a pure tone synthesized with a linear offset, (**c**) *Clicks* (left) and *Click trains* (right), (**d**) *OMAR* stimuli such as a dog barking, chicken clucking and bird chirping, and (**e**) *SESAME* stimuli including an amplitude modulated tone, two pedestal tones, a speedbump tone, rising tone.

We initially grouped sounds into one of five categories based on the descriptions given in the article and online links: (i) *flat*, (ii) *percussive*, (iii) *click train*, (iv) *other*, and (v) *undefined*. Our “flat” category included sounds with a period of invariant sustain and defined rise/fall times, such as “a 500-Hz sinusoid, 150 msec in duration…gated with a rise-decay time of 25 msec”^44^. Similarly, we classified sounds described as “rectangularly gated”^45^, having a “rectangular envelope”^46^, “trapezoidal envelope”^47,48^, “square-gate”^49^, “fade-ins and fade-outs to avoid clicks”^50^ or “abrupt onsets and offsets”^51^ as flat. Samples of sounds falling into this category appear in the top row of Fig. 2.

Our second category, “percussive,” encompassed sounds with sharp onsets followed by gradual decays with no sustain period (i.e. impact sounds). This included sounds from cowbells^52^, bongos^53^, drums^54^, chimes and bells^55^, marimbas^56^, vibraphones^57^, and pianos (in which hammers impact strings)—both natural^58^ and synthesized^52,59^. Environmental impact sounds such as hand claps^55^, footsteps^60^, dropped^61^ and struck objects^62,63^ also fell into this category. In addition to natural sounds, this category included synthesized tones with ‘damped’ envelopes^64–69^. For example, we considered a “target tone (5-ms rise time)…[that] terminated with a 95-ms linear offset ramp”^68^ to be a percussive ‘damped’ tone. Waveforms of stimuli categorized as percussive are shown in the second row of Fig. 2 and are summarized in detail in Supplemental Table 1.

Our third category of “click/click train” contained sounds described as clicks or a series of repeated stimuli over a short duration (refining our earlier approaches^39^). This included sounds explicitly identified as “clicks”^70,71^ or “transients”^72^, as well as as “click trains”^73^, “pulse trains”^74,75^, “pulses in a train”^76^, or stimuli “presented in rapid, successive bursts”^77^. We also included click trains of variable rates^78^ within this category (see third line of Fig. 2 for examples).

Our fourth category of “other” initially contained all sounds with defined amplitude envelopes other than those previously described. We subsequently split this category based upon referentiality—whether or not the sounds originated from real world events. Referential sounds included environmental sounds^79–81^, recordings of animals such as dogs and/or chickens^54,55^, and collections of sounds such as those heard at bowling alleys, beaches, and construction sites^54^. This also included a variety of non-percussive musical sounds such as brass^55,82,83^, string^81,82^, and woodwind instruments^57,84^, including instrument sounds later shortened^85,86^ or filtered^82^. Additionally, excerpts of popular music^87^ as well as choral singing^88^ fell into this category. We named this new group *OMAR* as it encompassed Other Musical And Referential sounds (i.e. referential sounds other than those included in the percussive category). Despite its broad nature this category ultimately contained the smallest percentage of sounds (fourth row of Fig. 2).

The *other* category also included non-referential sounds, i.e. those lacking a real-world referent. This includes amplitude modulated tones^89^, pedestal tones^90,91^, tones with defined rise/fall times and no sustain; both symmetric (e.g. 50 ms rise/fall time)^92–95^ and asymmetric (e.g. 15 ms rise 45 ms fall)^96^, as well as reversed-damped or ‘ramped’ tones^64,66,68,69,97^. We named this subcategory *SESAME*—Sounds Exhibiting Simple Amplitude Modulating Envelopes. These sounds include some amplitude variation beyond onset/offset, yet lack real world referents (note that although rising tones are often regarded as mimicking approaching sounds^35,36^, this only holds if the approaching sounds are flat^98^). Although this category’s definition is somewhat broad, it ultimately contained the second fewest number of stimuli (after OMAR). Depictions of these stimuli appear in the final line of Fig. 2, and Supplemental Table 1 provides a detailed breakdown of sounds classified under this category.

Finally, we used a fifth category of “undefined” for sounds whose amplitude envelopes could not be discerned from the information provided. For example, we classified the amplitude envelope of sounds described as ‘a 500 ms, 1000 Hz tone’ as undefined. We treated this as a category of last resort, using it only when unable to discern any information regarding temporal structure. For example, when authors stated they used stimuli defined in other papers^99–102^ or included links to online repositories^55,103^, we obtained and analyzed the supplementary information. This avoided labeling stimuli as undefined when authors had merely been judicious with space.

### Definition of six crucial properties

We also coded stimulus duration, as well as the presence or absence of information on additional characteristics such as spectral structure and intensity, and technical equipment details such as delivery device (i.e. headphone/speaker) and tone generator make/model. This expanded our team’s previous approach^39^ of classifying these properties only for stimuli with undefined amplitude envelopes.

We created three categories for coding these properties: Specific, Approximate or Undefined (see Table 4 for examples). For example, we coded the intensity of stimuli described at “70 dB” as *Specific*, those “at a comfortable level” as *Approximate*, and those lacking any information on intensity as *Undefined*. Similarly, we coded delivery device information of “Sennheiser HD265 headphones” as *Specific*, general mention “headphones” as *Approximate*, and the lack of any information about sound delivery as *Undefined*. This helps contextualize our exploration of amplitude envelope by providing useful comparators for levels of definition of five other properties.

**Table 4:** Examples of Undefined, Approximate and Specific Descriptions of Properties.

Property	Undefined	Approximate	Specific
Amplitude Envelope	No information provided	Trapezoidal envelope, MIDI Cowbell, Recording of bird song, Click train	25 ms rise/fall time and 200 ms sustain, Train of five rectangular pulses (0.2 ms wide, 10 ms interval) presented bilaterally every 2 s
Spectral Structure	Tone, Signal, Stimulus	500 Hz Tone (i.e. tone with specified fundamental)	Sinusoid/Pure Tone, Broadband White Noise, Piano, Bird Chirp
Duration	No information provided	Continuous, Variable, 60 ms-1000 ms (range)	400 ms, 1.5 s, 20 μs
Intensity	No information provided	Comfortable listening level, 30 dB above absolute threshold	70 dB SPL, 88 dB
Delivery Device	No information provided	Headphones Loudspeaker Computer monitor speaker	TDH-49 headphones, Sennheiser HD265 headphones, Sony Speakers (Model SRS-A91)
Sound Generator/Source	No information provided	Digital recordings of Instruments, Pure-tone oscillator, 8-bit digital-to-analog converter	Grason-Stadler 455 C noise generator, IBM Thinkpad 560X with an audiocard (TDK DMC9000), Hewlett-Packard Model 200 ABR oscillator

## Results and discussion

**Figure 3:**
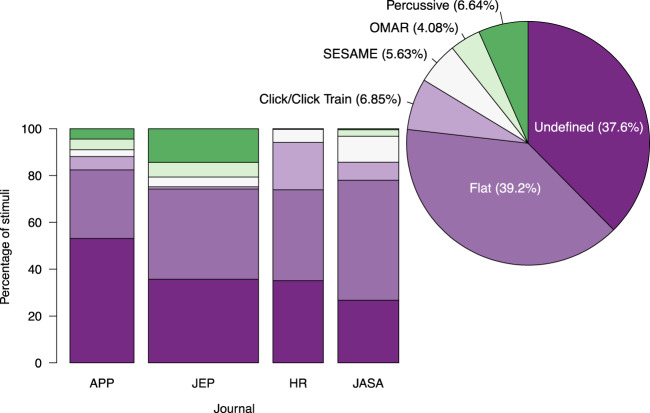
Amplitude envelope distribution. Bars indicate distribution within each journal, with width indicating the journal’s relative points. JEP contained more multi experiment papers and therefore contributed the most points (see Table 2 for a detailed breakdown). Pie chart shows the grand summary across all stimuli.

**Figure 4:**
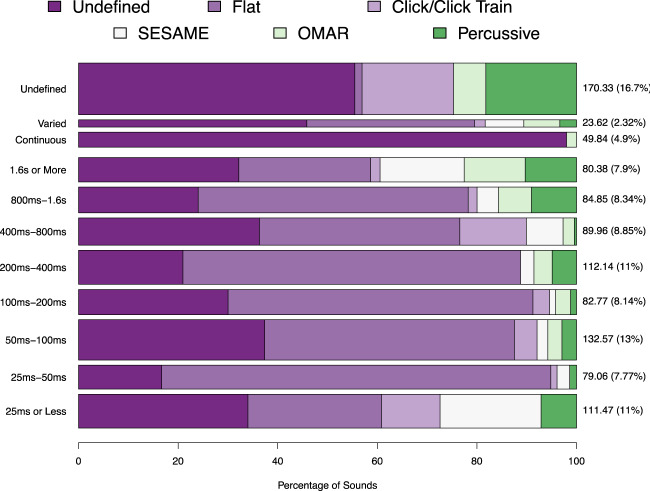
Distribution of stimuli by duration. The lack of definition is not confined to short sounds. The lowest row groups stimuli less than 25 ms, with each row doubling in duration. The top three rows indicate envelope distribution for stimuli with undefined durations (~17% of observed stimuli), as well as those with defined durations that varied, or sounded continuously (i.e., background noise). Bar width reflects relative number of points, with specific points (and percentages of total points) to the right of each bin.

Our analysis illustrates a surprising lack of attention to the reporting of amplitude envelope, with 37.6% of stimuli from 1017 experiments omitting any information about their temporal structure (Fig. 3). This varied somewhat by journal: 53.1% (APP), 35.7% (JEP), 35.1% (HR), 26.9% (JASA), providing useful perspective on our team’s previous survey of the journal *Music Perception*, which fell within this range^39^. As the lack of definition is fairly consistent across duration categories (Fig. 4), it is not driven by the use of extremely short sounds in which amplitude changes would be imperceptible.

To contextualize the under-reporting of amplitude envelope, we compared its definition to that of other stimulus properties (spectral structure, duration, and intensity), as well as technical equipment information—such as the exact make and model of delivery device (e.g., Sennheiser HD265 headphones, Sony SRS-A91 Speakers) and sound generating equipment (e.g., Grason-Stadler 455 C noise generator, Hewlett-Packard Model 200 ABR oscillator) used. As shown in Table 5, we observed significantly less detail about amplitude envelope than most surveyed properties. Authors omitted duration information for only 16.7% of stimuli, and spectral structure for a mere 4.1%. This contrasts with amplitude envelope’s lack of definition for 37.6% of stimuli—the highest of all properties surveyed. Curiously, we found authors significantly more likely to include the *exact model* of delivery device than *any information* about amplitude envelope (χ2 = 5.87, p = 0.015).

**Table 5:** Definition levels of six properties. All other properties of sound coded were defined at significantly higher rates than amplitude envelope.

*Comparisons of defining amplitude envelope vs*. *other properties*			
Property	Undefined Raw Points	Undefined Percentage	Chi Squared Test
Spectral Structure	42.15	4.1%	χ^2^ = 272.91, p < 0.001
Duration	170.33	16.7%	χ^2^ = 81.51, p < 0.001
Delivery Device	200.17	19.7%	χ^2^ = 57.12, p < 0.001
Intensity	204.38	20.1%	χ^2^ = 54.13, p < 0.001
Sound Generator/Source	353.00	34.7%	χ^2^ = 1.19, p = 0.275
Amplitude envelope	382.63	37.6%	N/A

### Interpreting the undefined tones (and illuminating the larger problem)

Although the lack of definition regarding amplitude envelope is surprising, we believe the more important issue illuminated by this suvey is the heavy focus on flat tones in non-speech auditory research. As shown in the grand summary of all four journals (pie chart in Fig. 3), flat tones formed the largest group in the survey—39.2% of sounds encountered. Clicks/Click trains formed the second largest group of defined stimuli (6.85%). Percussive sounds formed the third largest group (6.64%), followed by SESAME tones (5.63%) and OMAR sounds (4.08%). The use of flat tones outnumbered that of all other classifications combined—62.8% of defined stimuli. Furthermore, we strongly suspect that the vast majority of undefined stimuli are in fact flat.

Given the prominence of both the authors and journals surveyed, we find it unlikely that researchers neglected to disclose amplitude changes in their synthesized sounds. Additionally, based on feedback from conferences flat tones appear to serve as a go-to stimulus for assessing hearing, and we have often encountered surprise from colleagues when realizing that descriptions of a “short tone” could refer to anything else. Furthermore although their prevelance ranged considerably amongst journals, Fig. 3 shows remarkable consistency in “presumed flat” tones—a combination of the flat and undefined categories: 82.4% (APP), 74.2% (JEP), 73.9% (HR), 77.9% (JASA). For these reasons we strongly suspect that undefined tones are in fact flat. Therefore presumed flat tones constitute over three quarters (76.8%) of surveyed stimuli, with the majority of the remaning non-flat tones either Clicks/Click Trains or SESAME sounds.

### The role of temporal complexity and referential sounds

In the process of defining stimulus categories for this project, we realized the utility of grouping sounds based on their referentiality—whether they refer to physical events. Both Percussive and OMAR sounds (Fig. 2) originate from real-world events outside the laboratory. Percussive sounds are created by musical instruments (drums, pianos) or natural impacts such as footsteps^60^, as well as synthesized tones mimicking receding^69^, departing^66^ damped^64^ or “dull”^68^ sounds. OMAR sounds include musical tones produced by blowing or bowing (including synthesized versions), as well as soundscape recordings of the beach and/or forest^54^ and specific events such as animal vocalizations^80,83^, and water poured into a glass^54^. We also consider sounds produced by helicopters^79^ trains^55^ and car engines^104^ to be referential, as they are derived from physical events.

Despite its broad definition, only 10.7% of the total stimuli encountered are referential (20.7% JEP; 9.0% APP; 3.2% JASA; 0.3% HR). Therefore 89.3% of these auditory stimuli have no connection to real-world events. As this sample is likely representative of non-speech auditory perception research as a whole, we consider this an important insight, given that everyday listening is so grounded in its utility for understanding the environment—such as using sound to inform our understanding of objects and events^4,5^.

### How have stimulus selections changed historically

**Figure 5:**
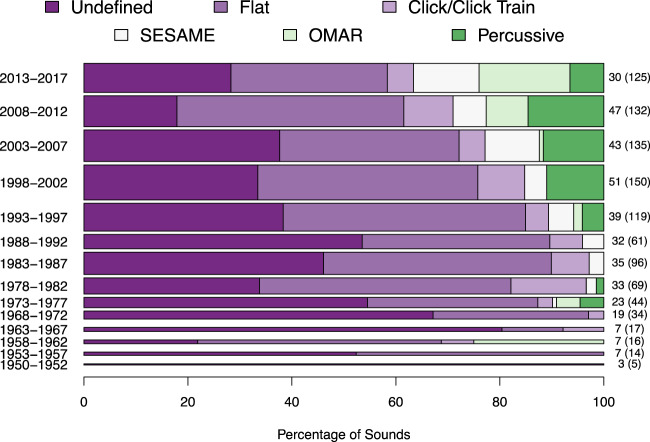
Changes in stimulus distribution over time. Researchers have used more diverse sounds in recent decades. However, note that even in the latest time bin, over half of stimuli surveyed are either flat or presumed flat, and less than 25% use referential sounds. Bar width indicates number of points associated with a given bin. Specific information on the number of papers appears to the right, with the number of points derived from these papers (i.e. the total number of experiments) in parenthesis. The earliest years are more sparsely sampled in part as they contain only JASA prior to 1966.

In order to examine changes in stimulus selection over time, we grouped our data into five-year bins starting in 2017 and going back to 1950 (Fig. 5). This illustrates growth in the use of referential sounds, particularly in the last two decades. Although encouraging, it indicates less an embrace of complex sounds than a *broadening of research questions*. For example, this includes a 2013 study of how music affects tinnitus^87^, a 2015 exploration of how airplane sound affects the taste of food^105^, and a 2015 study of how street noise affects perception of naturalistic street scenes^106^. Other tasks with referential sounds include a 2009 study of animal identification^55^, and a 2008 study of identifying a walker’s posture^60^. Therefore this increased use of referential sounds appears to indicate an expansion of the types of questions investigated, rather than a reassessment of basic theories and models derived, tested, and refined with an overwhelming focus on temporally constrained stimuli.

### Conclusions and Implications

Amplitude envelope’s significance^23^ in explaining why a novel audio-visual illusion breaks with accepted theory^16^ sparked our interest in understanding its importance in other aspects of auditory processing. Our team’s findings regarding its role in audio-visual integration^16,17,19,107^ duration assessment^26^, musical timbre^108^, associative memory^109^, and even perceived product value^110^ complement a growing literature with others documenting its importance in perceptual organization^24,25,111^, as well as evaluations of event duration^27–31^, loudness^32–34^, and loudness change^35,36^. Together, these studies suggest that research focused heavily on flat tones might overlook and/or misrepresent the capabilities and capacities of the auditory system. In several instances their disproportionate use has demonstrably led to faulty conclusions—for example misunderstanding the role of vision in duration estimation^16,17,19,107^.

Despite long-standing speculation amongst leading figures in auditory perception^5,10^ and explicit notes of concern in the literature^11,12,112^, to the best of our knowledge there has not previously been a detailed survey of this nature. Consequently our examination of over one thousand auditory experiments from four highly regarded journals offers three insights of broad relevance: (1) under-reporting of amplitude envelope, (2) defaulting to the use of flat tones for non-speech research, and (3) relatively little attention to the importance of referential aspects of sounds. We will now discuss each point in turn, placing them in the context of ongoing areas of inquiry.

### Lack of attention to the reporting of amplitude envelope

The lack of attention to the reporting of amplitude envelope is our most surprising outcome. Well-respected authors publishing in highly regarded journals neglected to define amplitude envelope for 37.6% of stimuli. It is one thing to find a particular property to be under-researched; it is quite another to realize its importance has been so underappreciated that manuscripts fail to convey information about it in over one third of prominent auditory experiments. Although some may argue that descriptions such as “a 500 ms tone” imply flat tones, this ambiguous description fits a wide range of sounds. For example, all of the SESAME and flat stimuli shown in Fig. 2 are in fact 500 ms tones.

This lack of definition does not result from mere technicalities such as the prominence of very short tones (Fig. 4), or general inattention to methodological detail (Table 5). Curiously, our data suggest authors, reviewers and editors gave more emphasis to definition of the exact model of headphones used to deliver tone beeps, clicks, and bursts than any information regarding amplitude envelope. As every article included in this survey passed peer review in highly regarded journals, we see this oversight less as a failing of individual papers than as a cautionary note for the discipline as a whole. Among other concerns, this observation raises important questions regarding best scientific practice as researchers replicating these studies would in theory lack information needed to definitively recreate the sounds used. Our goal in clearly articulating this oversight is not to dismiss previous insights into the the auditory system, but merely to draw attention to the fact that this is an area in which we can improve as a discipline. Science progresses through critical reflection leading to refinement of best practices, and we are hopeful this survey will spark useful discussions about documention in future research studies.

Encouragingly, we note a slight increase in the amount of specification of amplitude envelope over time, with fewer undefined stimuli in more recent years (Fig. 5). We are hopeful this trend will continue, as definition of this property can only help to further clarify our understanding of its important role.

### Challenges with the use of flat tones as a default stimulus

More important than the lack of definition is the fact that flat tones account for over three quarters (77%) of stimuli encountered (when treating undefined tones as flat). As the survey drew upon on a representative selection of auditory research from four major journals, we believe this is indicative of standard approaches to auditory perception research. Flat tones hold certain methodological benefits such as avoiding potential confounds from associations with referential sounds, offering tight control, and/or minimizing variation between research teams. However, as they are processed differently than temporally varying sounds in a variety of contexts^24–36,107,109–111^ they should not be assumed to fully assess the limits or even the basic capabilities of the auditory system. Consequently, an over-reliance on flat tones poses serious problems for building a generalized picture of the auditory system’s capabilities.

To draw a lesson from other areas of perceptual inquiry, visual researchers have long recognized that we cannot fully appreciate object recognition by assessing vision using only static, 2D images^113^. Although unmoving stimuli are methodologically convenient (simple to generate and easier to equate than moving images), overreliance on them overlooks the crucial importance of movement^114^. Consequently, a full understanding of the visual system requires stimuli exhibiting cues posing challenges for experimental control. In many ways temporal variation in amplitude is “auditory movement,” and previous research documents that amplitude envelope plays an important role in signalling both the materials involved in an event^6,7^ as well as the event’s outcome. For example, amplitude envelope is helpful in understanding whether a dropped bottle bounced or broke^8^, as well as in determining an object’s hollowness^115^. Research focused disproportionately on sounds lacking the kinds of complex dynamic properties found in natural sounds may overlook crucial aspects of auditory processing—much as visual research using only static images can overlook motion’s role in visual processing.

The literature on duration assessment provides a useful example of potential problems arising from the overuse of flat tones (beyond numerous previously discussed examples in audio-visual integration). As mentioned in the Introduction, research on SET (Scalar Expectancy Theory)^37,38^ explores the perceptual processing of duration, positing in essence the use of a marker strategy– marking tone onset and offset and calculating the difference. However this strategy would be ill-suited for sounds with decaying offsets, which might instead be processed with a prediction strategy estimating tone completion from decay rate. A direct experimental test of duration assessment strategies found evidence consistent with the idea that different underlying strategies are used for sounds with flat tones and sounds with natural decays^26^, which might help explain why flat tones elicit different experimental outcomes than sounds with time varying amplitude envelopes in various perceptual organization tasks^23,25^. Although further research is needed to fully explore the issue, a bias towards the use of flat tones in assessing SET could lead to problematic situations where numerous experiments converge on and confirm one particular theoretical perspective for duration processing—which then fails to explain how duration is actually processed in natural sounds which often lack abrupt offsets.

### Problems with the pervasive nature of non-referential sounds

In many ways we see the most important outcome of this survey to be that so few non-speech auditory stimuli—just over 10%—emerge from real world events. Intriguingly, closer exploration of these referential sounds reveals that the vast majority are used in experiments *requiring real-world referents*. For example, studies exploring the recognition of animal vocalizations^55^, how street noise affects perception of street scenes^106^, and whether a walker’s posture can be identified by their footsteps^60^ simply could not be conducted without animal vocalizations, street sounds, and walkers’ footsteps respectively. Studies using referential sounds for traditional tasks such as sound localization^85,86^ and auditory-haptic interactions^58^ constitute only a small fraction of the 10.7% of referential sounds encountered.

It appears that non-referential (and in particular flat) tones serve as the default auditory stimuli for non-speech research. Tone beeps, clicks and SESAME tones are used for the vast majority of research on core theoretical issues, such as the perception of loudness^32–34,116^ and duration^117^ as well as sound-in-noise detection^48,89,118^ localization^119^, and stream segregation^120,121^. This raises important questions about the stimuli best suited for exploring auditory processing—for although beeps and clicks offer precise control, the lack of real-world referents presents the perceptual system with sounds that differ in crucial ways from those encountered outside the lab^108^.

Given that the perceptual system evolved in an environment where sounds emanate from events (i.e. rocks falling) and actors (i.e. animal vocalizations), the disproportionate use of non-referential sounds in its assessment can lead to problematic conclusions regarding fundamental processes. For example, research on the ‘unity assumption’^122^ and/or ‘identity decision’^123^ explores the degree to which the kinds of supra-modal congruence cues pervasive in natural events affect cross-modal binding, a process essential for our ability to function in a multi-sensory world. This includes but is not limited to semantic congruencies^124,125^, synesthetic correspondences^126^, and learned associations between arbitrarily-paired stimuli^127^. Understanding binding in this context requires the use of co-occurring sights and sounds (which are by definition referential). As this makes the tight control desirable for experiments challenging, research on the unity assumption serves as a useful domain for illustrating problems with the relative paucity of naturalistic sounds used in psychophysical experiments.

To apply controlled methodology to a domain that has long been studied with less rigorous methods, Vatakis and Spence documented stronger integration of gender-matched (vs. mis-matched) faces and voices, providing important evidence for the unity assumption in a tightly controlled psychophysical context^124^. Subsequent expansions assessed whether non-speech events could trigger the unity assumption—such as notes played on the piano vs. classical guitar^128^. They found videos of a piano key being depressed integrated similarly with the sound of a piano as well as a guitar (and that the guitar plucking gesture also integrated similarly with both sounds). Vatakis and Spence interpreted these data as indicating that event unity (i.e., the pairing of gestures and sounds emanating from the same event) had no meaningful effect on multi-modal binding. These outcomes along with others using non-musical impact sounds such as noises from objects being struck vs. dropped^128^ and vocalizations by humans vs. monkeys^129^ led to their conclusion that the unity assumption did not extend beyond speech.

Curiously, Vatakis and Spence’s experiments overlooked the crucial role of amplitude envelope. Notes produced by the piano and guitar share similar temporal structures, with a sharp attack and immediate decay resulting from either a hammer striking a string (piano) or the plucking of a string (guitar). Our team replicated their paradigm using notes from instruments with different amplitude envelopes—either percussive (marimba) or sustained (cello). In doing so, we found clear evidence for the unity assumption in a non-speech task^107^, in contrast to its absence in a similar task involving piano/guitar pairs^128^. This discrepancy is consistent with a broader literature on the importance of cross-modal congruency in the binding of impact sounds—particularly with respect to the role of amplitude envelope^17,19,25,111^.

Although oversight of amplitude envelope’s crucial role in the unity assumption by an internationally renowned research team is surprising, it is consistent with the relative lack of attention to natural sounds. If only ~10% of stimuli have real-world referents, it is understandable that important distinctions within this category have gone overlooked. This illustrates one challenge with disproportionately using non-referential stimuli such as beeps, buzzes, and clicks. Sounds with temporal variations constitute the majority of our everyday listening—as well as the entirety of our evolutionary history. Yet they appear to be avoided whenever possible in basic non-speech auditory perception research. Although their complexity comes with obvious challenges, avoiding them risks overlooking the ways in which this same complexity is routinely and effectively used by the auditory system in basic processing—similar to problems using only static stimuli to understand object recognition^114^ which are gaining increasing attention in visual research^113^.

### Final thoughts

Although most relevant to those working in audio-visual integration, there are at least three reasons why this survey holds important messages for the field of auditory perception as a whole. First, amplitude envelope is recognized as playing a role in shaping perception of musical timbre^13–15,108^ as well as duration^26–31^ loudness^32–34^, loudness change^35,36^ and even associative memory^109^. Consequently there is good reason to believe its importance could extend widely beyond the context in which it has been most clearly shown to play a role—audio-visual integration^16,17,19,24,25,107,111^. Second, further evidence of amplitude envelope’s effects on key theories and models can only be discovered by recognizing the value of broadening our stimulus toolset. As contemporary sound synthesis programs can easily faciliate the precise generation of tones with more amplitude variation^130^, the primary barrier to their use is no longer technical but historical—choosing flat tones by default. Consequently this survey illustrates trends difficult to observe from any single experiment, and provides unique insight into challenges with current approaches. Third, the use of time varying envelopes holds tremendous immediate potential for use in applied work. For example the International Electrotechnic Commission mandates the use of flat tones in many auditory alarms^131^, which is one (of several) well documented problems^132,133^. Alternative amplitude envelope shapes can improve their suitability for wide-spread use^134^ yet have been rarely explored to date. Therefore efforts to raise awareness of this issue are pertinent for the auditory community as a whole, and for projects both theoretical and applied. To aid with this issue we have also created an online tool offering interactive visualizations of our survey data at www.maplelab.net/survey.

In conclusion, we strongly encourage both (a) the greater specification of amplitude information and (b) the use of a more diverse stimulus set in future studies. To be clear, we do not think flat tones should be avoided entirely, nor should non-referential tones be eliminated from our repertoire. Both offer certain benefits, and in some situations are adequate or even ideal—particularly when a lack of previous associations is desirable. Our concern is not that such sounds are used in auditory research, but rather that they are *used so disproportionally*. Greater consideration of how experimental outcomes might vary with sounds exhibiting natural amounts of temporal complexity would help address concerns from leading researchers that the world is “[not] replete with examples of naturally occurring auditory pedestals [i.e., flat amplitude envelopes]”^11^ and that more attention is needed to sounds with amplitude envelopes “closer to real-world tasks faced by the auditory system”^12^.

## Supplementary information

Supplemental table.

## Data Availability

The datasets generated during and/or analysed during the current study are available from the corresponding author on reasonable request.
