# Supplementary material for: On the generalization of tones: A detailed exploration of non-speech auditory perception stimuli
Source: Sci Rep. 2020 Jun 12;10:9520. doi: 10.1038/s41598-020-63132-2 (PMC7293323; doi:10.1038/s41598-020-63132-2)
Supplement: Supplementary file 1 — Supplemental table. [file 41598_2020_63132_MOESM1_ESM.pdf]

On the generalization of tones: A detailed exploration of non-speech auditory perception stimuli

Michael Schutz* [1,2,] Jessica Gillard[2,]

[1] School of the Arts, McMaster University, Canada

[2] Department of Psychology, Neuroscience & Behaviour, McMaster University, Canada

Supplemental Table 1. Breakdown of specific sound types encountered within each category. Second column gives representative description of sounds. Last two columns give the absolute number of points for the category, as well as percentage of overall point total. For compactness, subcategories under 2% of survey total are grouped into a single line. For example, we identified 5 points of Organ sounds, which fell under the Musical Instrument category (the Musical instrument category itself contained 15.8 points, approximately 1.55% of the total encountered in the survey). Note we classified several 400 ms snippets of drum, triangle, and guitar tones with 10 ms linear onset/offset ramps [85,88] under OMAR.

Detailed listing of sounds

Envelope	Subcategory	Point Weighting	% of Total Points
Undefined	-	382.13	37.6%
Flat	-	398.96	39.2%
Click/Click Train	-	69.67	6.85%
SESAME	Speedbump Tones	26.45	2.60%
(Sounds Exhibiting Simple Amplitude Modulated Envelopes)	Amplitude Modulated (13.17), Hybrid Flat-Damped (5), Pedestal Tone (3.67), Ramped (3.43), Blip (3), Pyramid (1.5), Misc. Synthesized Sounds (1)	30.77	3.03%
OMAR	Environmental	25.72	2.53%
(Other Musical And Referential)	Organ (5), Musical Excerpt (2.83), Woodwind (2.46), Brass (1.60), Percussion* (1.5), String (1.41), Singing (1)	15.80	1.55%
Percussive	Piano	28.67	2.82%
	Environmental	21.78	2.14%
	Percussion (8.45), Damped (7.33), Drum (0.56), Music (0.40), String (plucked) (0.31)	17.05	1.68%
**Total**		**1017**	**100%**
